# Traumatic physical health consequences of intimate partner violence against women: what is the role of community-level factors?

**DOI:** 10.1186/1472-6874-11-56

**Published:** 2011-12-20

**Authors:** Diddy Antai

**Affiliations:** 1 Division of Global Health & Inequalities, The Angels Trust - Nigeria, Abuja, Nigeria; 2 Karolinska Institute, Department of Public Health, Division of Social Medicine, Stockholm, Sweden

## Abstract

**Background:**

Intimate partner violence (IPV) against women is a serious public health issue with recognizable direct health consequences. This study assessed the association between IPV and traumatic physical health consequences on women in Nigeria, given that communities exert significant influence on the individuals that are embedded within them, with the nature of influence varying between communities.

**Methods:**

Cross-sectional nationally-representative data of women aged 15 - 49 years in the 2008 Nigeria Demographic and Health Survey was used in this study. Multilevel logistic regression analysis was used to assess the association between IPV and several forms of physical health consequences.

**Results:**

Bruises were the most common form of traumatic physical health consequences. In the adjusted models, the likelihood of sustaining bruises (OR = 1.91, 95% CI = 1.05 - 3.46), wounds (OR = 2.54, 95% CI = 1.31 - 4.95), and severe burns (OR = 3.20, 95% CI = 1.63 - 6.28) was significantly higher for women exposed to IPV compared to those not exposed to IPV. However, after adjusting for individual- and community-level factors, women with husbands/partners with controlling behavior, those with primary or no education, and those resident in communities with high tolerance for wife beating had a higher likelihood of experiencing IPV, whilst mean community-level education and women 24 years or younger were at lower likelihood of experiencing IPV.

**Conclusions:**

Evidence from this study shows that exposure to IPV is associated with increased likelihood of traumatic physical consequences for women in Nigeria. Education and justification of wife beating were significant community-level factors associated with traumatic physical consequences, suggesting the importance of increasing women's levels of education and changing community norms that justify controlling behavior and IPV.

## Background

Intimate partner violence (IPV) - "any acts of physical, sexual or emotional abuse by a current or former partner whether cohabitating or not" [1] - is an important cause of morbidity and mortality [2], with adverse effects for women's physical, mental, sexual and reproductive health [2-4]. The relationship between IPV and health is complex; the consequences may be immediate and direct (such as injury or death), longer term and direct (such as disability), indirect or psychosomatic (such as gastrointestinal disorders) or all three [5]. While direct effects such as death are beyond the scope of this study, evidence shows that about half of the women in abusive relationships sustain physical injuries [6-8]; these injuries vary from minor to life threatening injuries. Minor injuries (scratches, bruises, welts) are most common, whilst others, such as lacerations, knife wounds, broken bones, head injuries, broken teeth, burns, and bullet wounds occur with decreasing frequency [9]. Studies of emergency departments in the USA and elsewhere suggest physical abuse as a major cause of injury in women [10,11].

### Theoretical framework

**Figure 1:**
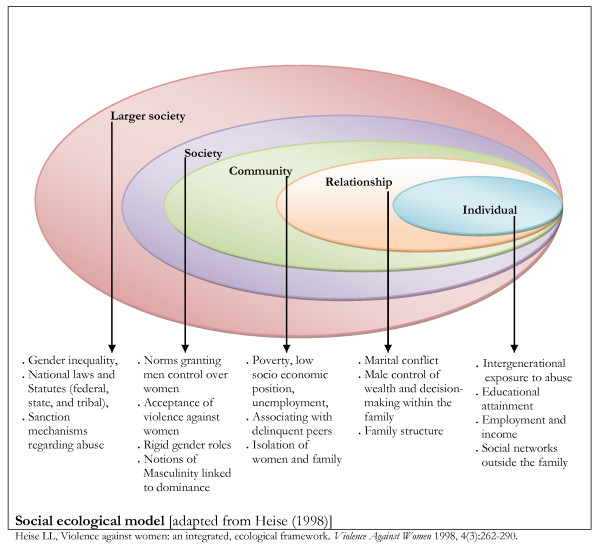
**Social ecological model**.

The socio-ecological model [12] used in this study provides a comprehensive understanding of factors that may influence IPV vulnerability, coping and consequences at different levels (Figure 1). Factors related to IPV victimization are identified at five levels [12,13]. The individual or intrapersonal level refers to individual experiences related to gender norms and values that predispose women to abuse [14], such as witnessing or being abused [15], or being financially dependent [16,17]. The relationship or interpersonal level refers to the interactions between couples, families and other small groups, such as male control over family resources, decision-making autonomy, economic inequalities, and high levels of controlling behaviour by husband or partner [17-19]. Controlling behavior by husband or husband has been shown to be associated with an increased likelihood of IPV, and reflects the increased vulnerability of abuse experienced by women in male-dominated family structures and social order; this encourage men to exercise control over women within patriarchal societies such as Nigeria [20-22]. Evidence has shown that husbands who believed men's superiority over women were more likely to be abusive [23], whilst a study in the United States showed a positive association between women's acceptant attitudes of wife beating and the occurrence of abuse [24]. Women's justification of traditional societal norms of wife beating by a husband/partner has been shown to be strongly correlated with different forms of IPV [20-22], and widely regarded to be a consequence of women's acceptance of such acts of violence as well as the socio-cultural factors permitting men to inflict punishment on their wife/partner. This level of societal response to partner violence may influence controlling behavior, and the likelihood of IPV with possible consequences [25].

Interpersonal dimensions of control and power can also be expressed by decision-making autonomy, and the ability to engage in actions against a partner's wishes [20]. Unequal distribution of power and economic dependence between partners in a relationship are key elements in control within relationships [26]. Given that individuals with less power are less equal to the partners with more power, they may be victims of control by the partner with more power. Thus, relationships of equally dependent partners that embrace egalitarian decision-making and an equal division of power within the family more often report low levels of conflict, control, and abuse [27,28]. Women who are defiant to societal gender roles may be regarded as challenging their partner's masculinity as provider or breadwinner, thereby becoming vulnerable to their partner's control tactics to curtail such defiance, which may result in abuse [29]. Dimensions of relationship inequality focused on in this study include spouses' relative earning, spouses' relative education, and spouses' relative age. In certain societal contexts like the one under study, the greater the equality (or less inequality) between partners, the higher the women's risk of exposure to abuse as this threatens certain men's position of power [30,31]. For example, women whose economic resources approach or exceed that of their partners' resources tend to be more vulnerable to control and abusive acts [32].

The community level within the socio-ecological model focuses on the relationships of members of a specific physical or psychological community or neighbourhood, such as the high level of IPV in societies where violence is the norm [33,34]. Finally, the societal level focuses on the dominant societal norms, national laws and statutes (federal, state, and tribal) and lack of adequate sanction mechanisms regarding abuse [35].

In summary, this model indicates that causes and outcomes of IPV reflect the interplay between factors at multiple levels. In as much as individual vulnerability to IPV may easily be linked with factors at the individual and interpersonal levels, they may also be traced to factors operating at the higher levels of society, institutions, communities, and social policy. Thus, the ecological model offers a comprehensive public health approach for prevention and intervention measures for IPV and health consequences [36]. Despite a well-documented association between place of residence and violent victimization [37], most of the existing literature focuses on individual-level determinants of IPV [38,39], whilst often failing to recognize the multidimensional nature of the determinants of IPV, especially the role of community-level factors. Examining the role of communities in the likelihood of health consequences following IPV may be especially pertinent considering that the use of violence is normatively accepted in several countries, though there is a clear limit to the tolerated severity of violence [35]. Internalization of these norms by women themselves in patriarchal societies may result in women believing and thereafter justify a man's right to beat his wife under certain circumstances, such as when she goes out without telling him, neglects the children, argues with him, refuses to have sex with him, or she burns the food [16][40]. Evidence from a socio-ecological analysis of DHS data from 17 countries indicated that women were twice as likely as men to justify physical violence [41]. The present study examines how characteristics of the communities in which women reside in influence their exposure to IPV and traumatic physical consequences. The aims of this study were therefore to explore the characteristics and magnitude of the range of traumatic physical health outcomes associated with physical IPV, and to assess the role of key contextual factors associated with these consequences.

#### The Nigerian context

Nigeria is Africa's most populous nation with a population of 140 million inhabitants. It is extremely heterogeneous, with geographical, cultural and ethnic diversity. There are about 374 identifiable ethnic groups, with the Hausa, Yoruba, and Igbo being the major ethnic groups. 50% of Nigerians are Muslims, 40% are Christians and 10% have traditional indigenous beliefs. Nigeria is made up of 36 states and a Federal Capital Territory (FCT), which are grouped into six geopolitical regions: North Central, North East, North West, South East, South South, and South West. IPV is not unique to Nigeria; however it is pervasive within the country [42-44]. The traditional Nigerian society is patriarchal, with gender roles (social norms about the proper roles and responsibilities of men and women - being skewed to justify violence against women who are assigned an inferior role to men [42,43]. These gender roles are deeply rooted, institutionalized, and endorsed as means of settling domestic conflicts. For instance the *Sharia *Penal Code (laws that define the elements of particular crimes and specify the punishment for each crime), which is applied only in the predominantly Muslim northern part of the country, permits husbands to 'correct' their wives as long as such correction does not result in grievous harm, in this case defined as loss of sight, hearing, power of speech, facial disfigurement or life-endangering injuries [45,46].

Typical of the patriarchal social structure, gender roles tend to be strictly defined and adhered to within traditional societies, as evidenced in the practice of bride price [47], and polygyny (a normative marital system in many African societies that structures social relationships within the household by placing the co-wives in direct competition with each other, all the while being under the authority of a husband) [48,49]. The other cultural norms acceptance of wife beating, which reflects women's endorsement of cultural beliefs that permit men to use violence to control their partner [47] increase the tendency for men who subscribe to male superiority over females to perpetuate abuse.

### Hypotheses

This study hypothesizes that: i) IPV will be associated with a higher likelihood of traumatic physical consequences even after adjusting for other individual- and community-level factors; ii) controlling behavior by husband/partner will be associated with increased likelihood of IPV, and thus traumatic physical consequences, given that controlling behavior reflects a tendency to exercise power over a partner, as seen in recent evidence [20,21]; iii) women who justify wife beating will have a higher likelihood of experiencing IPV and traumatic physical consequences; this hypothesis is based on the assumption that women who justify wife beating are more likely to tolerate acts of abuse from men who adhere to traditional gender roles [50], and men who use force to ensure women's compliance resulting in IPV and traumatic physical consequences; and iv) women with decision-making autonomy will have a higher likelihood of experiencing IPV, given that women's greater assertiveness may derive from greater decision-making autonomy, which may deviate from cultural norms, and cause certain men to perceive themselves as less dominant or less assertive than their wives. Such men may in turn resort to violence to maintain dominance [51,52], which may result in traumatic physical consequences.

## Methods

### Design

Cross-sectional data from the 2008 Nigeria Demographic and Health Survey (DHS) was used in this study. Details of the study design are discussed elsewhere [53]. Briefly however, data were collected using a stratified two-stage cluster sample design. From the list of enumeration areas (EAs) developed from the 2006 Population Census sampling frame, 888 primary sampling units (PSUs) were selected in the first sampling stage, and a probability sample of 7864 households were systematically selected from these PSUs in the second sampling stage. Data were collected by face-to-face interviews from 33,385 women aged 15-49 years within the selected households resulting in a nationally-representative sample. Permission to use this data in this study was obtained from ORC Macro Inc. and approval obtained from the National Ethics Committee in the Federal Ministry of Health of Nigeria and the Ethics Committee of the Opinion Research Corporation Macro international, Inc. (ORC Macro Inc., Calverton, MD; USA). Communities in which participant resided were identified within DHS data as PSUs; these are small and fairly homogenous administratively-defined areas representing sampling blocks of about 20 to 30 households.

### Participants

Of the total women (N = 33,385) within the 2008 NDHS survey, 23,752 (71%) women who were currently or formerly married or cohabiting with a male partner were selected and interviewed about domestic violence. Of these, 4162 women (17%) had ever been exposed to any IPV. The DHS domestic violence questionnaire was used to collect data from the selected women in accordance with the World Health Organization's ethical and safety recommendations for research on domestic violence [54]. This ensures women's safety, maximizes disclosure of actual violence by providing adequate training and support to field workers, ensuring that informed consent is obtained, and guaranteeing the privacy of respondents are the aims of the recommendations. The DHS domestic violence questionnaire is based on a modified and previously validated version of the Conflict Tactics Scale (CTS) [55].

### Outcomes

*Traumatic physical consequences *were the health outcomes of interest, assessed using answers to questions asked the women (e.g. "ever had bruises because of husbands act?") regarding the lifetime history of the following indicators: *i) *bruises; *ii) *injuries, sprains, dislocations or burns; *iii) *wounds, broken bones, broken teeth or other serious; and *iv) *severe burns.

### Exposures

*Lifetime abuse (or any IPV)*, was the primary exposure variable of interest, and was derived from responses to questions asked respondents about whether they had ever experienced one or several of the following acts of abuse by a current or former husband or intimate partner, such as: *i*) pushing, shaking or throwing something at her; *ii*) slapping her or twisting her arm; *iii*) punching or hitting her with something harmful; *iv*) kicking or dragging her; *v*) strangling or burning her; *vi*) threatening her with a weapon (e.g. gun or knife); *vii*) attacking her with a weapon; *viii*) humiliating her in public; *ix*) threatening her or someone close to her; *x*) forced sexual intercourse; and *xi*) other sexual acts when undesired. Lifetime abuse (any IPV) was determined by any physical, sexual or emotional violence using the 11 items (*i - xi*); Cronbach's alpha (α) was.811.

Other measures of relationship control included: *i) controlling behavior*, were assessed as a composite dichotomous variable consisting of responses to six questions about whether present or former husband/partner had such control issues as: jealous if she talks with other men, accuses her of unfaithfulness, does not permit her to meet her friends, tries to limit her contact with family, insists on knowing where she is, and doesn't trust her with money. Women who responded "yes" to one or several of the control questions formed one group of the dichotomy, and the women that responded "no" to all the controlling attitude questions formed the other group of the dichotomy. Similar scale has been used in another study [56]. Cronbach's alpha for controlling behavior was .90; *ii) justifies wife beating*, a composite dichotomous variable created from responses to five questions regarding whether the women would justify abuse of a woman reasons such as when she goes out without telling him, neglects the children, argues with him, refuses to have sex with him, and burns the food. Women who responded "yes" to one or several of these attitude questions were grouped as "yes", and women who responded "no" to all the attitude questions were grouped as "no". Cronbach's alpha (α) was .88; and *iii*) *decision-making autonomy*, a composite dichotomous variable created from responses to 5 questions about whether the women had the final say regarding domestic decisions such as large household purchases, daily household purchases, visits to family or friends, own health, and deciding what to do with husband's money. Women who responded "respondent alone" or "respondent and husband/partner" to one or several of these questions were grouped as "yes", and those who responded "respondent and other person in the household" and "no" to all these questions were grouped as "no". The need for women to make decisions on common issues such as those referred to here with another person in the household (i.e. anyone other than her husband/partner, and most commonly mother in-law, husband/partner's relatives) on a daily basis is indicative of lack of decision-making autonomy. Similar grouping has been used in other studies [16][20][57]. Cronbach's alpha (α) for autonomy in domestic decisions was .89.

Variables reflecting relationship inequalities were: *i*) *spouses' relative earning*; *ii*) *spouses' relative education*; *iii*) *spouses' relative age*. Each of these variables was categorized as "same as husband/partner", "less than husband/partner", and "more than husband/partner" (including "woman's husband/partner does not contribute" in the case of relative earnings); and *iv*) *type of union*, categorized as monogamy (i.e. no other wife) and polygamy (i.e. ≥ 1 other wife). Demographic and socioeconomic variables included: *i*) *women's age*, grouped as ≤ 24, 25 - 34, ≥ 35 years; *ii*) *women's education *and *iii*) *partner's education*, both categorized as no education, primary education, and secondary or higher education; *iv*) *women's occupation *and *v*) *partner's occupation*, both categorized as professional, technical management; clerical, sales, skilled manual; agricultural self-employed, agricultural employee, household & domestic, unskilled manual; and not working (not working category was excluded in partner's occupation for having only 3 respondents); and *vi*) *place of residence*, categorized as urban and rural.

*Community-level measures of gender inequality *were operationalized as three indicators: *i) *community median age of first marriage, defined as mean age at first marriage for women in each PSU; *ii) community tolerant attitudes to wife beating*, defined as the percentage of women with tolerant attitudes to wife beating; and *iii) community mean education*, defined as the mean number of years of female education per woman in the community.

### Statistical Analysis

The association between outcome and exposure variables was assessed using cross-tabulations, with Pearson's chi-square (χ^2^) analyses (and Spearman's correlation for ranked variables); significance for all analyses was set at *p *< 0.05. Only variables significant at α = .10 in the bivariate association with IPV (Table not shown) were entered into the multilevel analyses. Two-level multilevel logistic regression models were fitted using generalized linear and latent mixed models (gllamm) [58], to account for the hierarchical nature of DHS data, and to examine the association between each traumatic physical consequence, any IPV, and other predictor variables. Respondents (at level 1) were nested within PSUs (at level 2). As the research objective was analytical and not descriptive, the unweighted data used in this study was justified, as recommended by Rutstein & Rojas (2003) [59]. The analyses were performed using Stata 11.0 [60]. Two models were fitted; model I contained IPV as the only variable to calculate the unadjusted effect of IPV, and model II included other individual and community-level or contextual variables to enable simultaneous examination of these variables and to assess whether factors at the community level exerted a contextual effect on the association between IPV and traumatic physical consequences. Results of fixed effects (measures of association) were expressed as odds ratios (OR) with 95% confidence intervals (95% CI). Random effects (measures of variation), which indicate the relatedness of clustered data were expressed as neighbourhood-level variance (σ^2^) with standard errors (SE). Intraclass correlation coefficient (ICC), which indicates the proportion of total variance that exists between neighborhoods i.e. level 2 [61] estimated the extent to which the propensity for traumatic physical health consequences for individuals within the same community was similar to that for individuals in other communities.

## Results

### Distribution of respondent characteristics

The lifetime prevalence of IPV was 17%. Bruises were the most common consequences (26%, n = 904) of IPV, followed by injuries, sprains, dislocations or minor burns (12%, n = 414), and less frequent were wounds, broken bones, broken teeth or other serious conditions (6%, n = 218), and severe burns (6%, n = 214). The proportion of the different traumatic physical health consequences sustained by IPV experience is presented in Table 1. The characteristics of respondents by experience of IPV (Table 2) showed that a significantly higher proportion of women who experienced IPV had a husband/partner with controlling behavior (3238 of 4162, 78%), justified wife beating (2425 of 4162, 58%), had decision-making autonomy (2844 of 4162, 68%), earned less than their husband/partner (2245 of 4162, 54%), and had the same level of education as their husband/partner (2363 of 4162, 57%). Most women who experienced IPV were 25 - 34 years old (1728 of 4162, 41%), had no education (1655 of 4162, 40%), were unemployed (3085 of 4162, 74%) and resident in rural areas (3078 of 4162, 74%).

**Table 1:** Proportion of traumatic physical consequences of by IPV exposure

	Bruises, N = 904		Injuries, N = 414		Wounds, N = 218		Burns, N = 214	
	
Characteristics	Yes, N (%)	No N (%)	Yes N (%)	No N (%)	Yes N (%)	No N (%)	Yes N (%)	No N (%)
*Intimate partner violence*	***p *<.113**		***p *<.026**		***p *<.014**		***p *<.008**	
Yes	809 (89)	310(12)	380 (92)	2684 (88)	201 (92)	2859 (88)	200 (93)	2864 (88)
No	95 (11)	2255(82)	34 (8)	371 (12)	17 (8)	392 (12)	14 (7)	391 (12)

**Table 2:** Distribution of respondent's characteristics by IPV exposure

	Any IPV		
		
Characteristics	Yes, N (%)	No, N (%)	*P*-value
*Controlling behavior*			*p *< 0.0001
Yes	3238 (78)	11103 (57)	
No	924 (22)	8487 (43)	
*Justifying wife beating*			*p *< 0.0001
Yes	2425 (58)	9384 (48)	
No	1737 (42)	10206 (52)	
*Decision-making autonomy*			*p *< 0.0001
Yes	2844 (68)	11531 (59)	
No	1318 (32)	8059 (41)	
*Spouses' earnings difference*			*p *< 0.0001
Woman earns less	2245 (54)	9522 (49)	
Woman earns same	612 (15)	3407 (17)	
Woman earns more	615 (15)	3290 (17)	
Woman's partner does not contribute	690 (16)	3371 (17)	
*Spouses' education difference*			*p *< 0.0001
Woman has less	598 (14)	3335 (17)	
Woman has same	2363 (57)	11129 (57)	
Woman has more	1201 (29)	5126 (26)	
*Spouses' age difference*			0.138
Woman younger	3815 (92)	15582 (80)	
Woman same age	166 (4)	2014 (10)	
Woman older	181 (4)	1994 (10)	
*Women's age*			*p *< 0.0001
≤ 24	910 (22)	5423 (28)	
25 - 34	1728 (41)	7480 (38)	
≥ 35	1524 (37)	6687 (34)	
*Women's education*			*p *< 0.0001
No education	1655 (40)	9143 (47)	
Primary school	1218 (29)	4584 (23)	
Secondary school or higher	1289 (31)	5863 (30)	
*Employment status*			*p *< 0.0001
Unemployed	1077 (26)	7720 (39)	
Employed	3085 (74)	11870 (61)	
*Place of residence*			
Rural	3078 (74)	12848 (66)	*p *< 0.0001
Urban	1084 (26)	6742 (34)	

### Determinants of traumatic physical health consequences

The association between IPV and traumatic physical health consequences is shown in Table 3. In the initial model, the likelihood of sustaining bruises (OR = 1.38, 95% CI = 1.02 - 1.85), injuries (OR = 1.64, 95% CI = 1.07 - 2.53), wounds (OR = 2.52, 95% CI = 1.29 - 4.91), and severe burns (OR = 2.78, 95% CI = 1.60 - 4.83) was significantly higher for women exposed to IPV compared to those not exposed to IPV. However, after controlling for other individual- and community-level factors in the full model, there associations were attenuated but remained statistically significant with the exception of injuries. Women who sustained bruises (OR = 1.75, 95% CI = 1.16 - 2.63), injuries (OR = 2.18, 95% CI = 1.36 - 3.51), wounds (OR = 2.93, 95% CI = 1.74 - 4.94), and severe burns (OR = 2.59, 95% CI = 1.46 - 4.62) were significantly more likely to be exposed to controlling behavior by husband or partner compared with those who were not exposed to controlling behaviour. Women who were 24 years or younger were significantly less likely to sustain wounds (OR = 0.45, 95% CI = 0.27 - 0.74) and severe burns (OR = 0.60, 95% CI = 0.36-0.99) in association with IPV compared to women who were 35 years or older. Sustaining severe burns following IPV was associated with a more than two-fold higher likelihood of women having no education (OR = 2.29, 95% CI = 1.33 - 3.95) and almost two-fold likelihood of women having primary education (OR = 1.87, 95% CI = 1.13 - 3.11) compared to women with secondary or higher education.

**Table 3:** Odds ratios and 95% confidence intervals for the association between intimate partner violence and traumatic physical consequences

	Bruises		Injuries		Wounds		Severe Burns	
	
Characteristics	Model 1	Model 2	Model 1	Model 2	Model 1	Model 2	Model 1	Model 2
	
	OR (95% CI)	OR (95% CI)	OR (95% CI)	OR (95% CI)	OR (95% CI)	OR (95% CI)	OR (95% CI)	OR (95% CI)
*Intimate partner violence*				-		-		-
Yes	1.38 (1.02-1.85)	1.91 (1.05-3.46)	1.64 (1.07-2.53)	1.85 (0.95-3.58)	2.52 (1.29-4.91)	2.54 (1.31-4.95)	2.78 (1.60-4.83)	3.20 (1.63-6.28)
No		1		1		1		1
*Controlling behavior*								
Yes		1.75 (1.16-2.63)		2.18 (1.36-3.51)		2.93 (1.74-4.94)		2.59 (1.46-4.62)
No		1		1		1		1
*Justifying wife beating*		-		-		-		
Yes								1.42 (0.96-2.11)
No								1
*Decision-making autonomy*		-		-		-		
Yes								0.79 (0.51-1.21)
No								1
*Spouses' relative earnings*						-		-
Woman earns less		0.71 (0.38-1.33)		1.10 (0.54-2.24)				
Woman earns same		1		1				
Woman earns more		0.86 (0.38-1.93)		1.14 (0.44-2.92)				
Woman's partner does not contribute		1.48 (0.65-3.37)		1.23 (0.47-3.22)				
*Spouses' relative education*				-				-
Woman has less		1.48 (0.28-7.78)				1.37 (0.51-6.22)		
Woman has same		1				1		
Woman has more		1.20 (0.85-1.68)				1.11 (0.73-1.57)		
*Spouses' relative age*		-		-		-		-
Woman younger								
Woman same age								
Woman older								
*Women's age*				-				
≤ 24		0.92 (0.58-1.46)				0.45 (0.27 - 0.74)		0.60 (0.36-0.99)
25 - 34		0.80 (0.57-1.11)				0.73 (0.52 - 1.02)		0.78 (0.53-1.15)
≥ 35		1				1		1
*Women's education*		-		-		-		
No education								2.29 1.33-3.95)
Primary school								1.87 (1.13-3.11)
Secondary school or higher								1
*Employment status*		-		-		-		-
Unemployed								
Employed								
*Place of residence*		-		-		-		-
Urban								
Rural								
*Community mean education*		0.85 (0.64-1.09)		0.74 (0.59-1.12)		0.91 (0.80 - 0.97)		0.83 (0.71-1.14)
*Community justify wife beating*								
Low		1.08 (0.67-2.42)		1.21 (0.52-2.41)		1.37 (0.73 - 1.88)		1.65 (0.38-2.42)
Median		1		1		1		1
High		2.16 (1.24-4.43)		2.02 (1.16-5.60)		2.70 (1.15 - 6.49)		2.69 (1.14-6.12)
*Community mean age of marriage*		1.06 (0.96-1.17)		1.04 (0.93-1.15)		1.17 (0.96 - 1.49)		1.13 (0.82-1.26)
**Random effects**								
Neighbourhood-level variance (SE)	0.376 (0.094)	1.936 (0.194)	0.600 (0.154)	0.494 (0.242)	1.491 (0.356)	1.507 (0.361)	1.379 (0.365)	1.433 (0.430)
Intra-class correlation (ICC)	0.103	0.370	0.154	0.130	0.312	0.314	0.295	0.303

^**a **^SE: Standard error

Women's education, operationalized as community mean education, was associated with a significantly lower likelihood of sustaining wounds (OR = 0.91, 95% CI = 0.80 - 0.97). Women resident in communities with high tolerance for wife beating had a higher likelihood of sustaining bruises (OR = 2.16, 95% CI = 1.24 - 4.43), injuries (OR = 2.02, 95% CI = 1.16 - 5.60), wounds (OR = 2.70, 95% CI = 1.15 - 6.49), and severe burns (OR = 2.69, 95% CI = 1.14 - 6.12) compared with women resident in communities with tolerance for wife beating at the median level for the community. The variance between neighbourhoods was more than twice the standard error in all the models, indicating significant differences between neighbourhoods in likelihood of bruises, injuries, wounds, and severe burns, respectively. The ICC in the full models (model 1) was 0.370 for bruises, 0.130 for injuries, 0.314 for wounds, and 0.303 for severe burns, indicating that 37% (bruises), 13% (injuries), 31% (wounds), and 30% (severe burns) of the total variance in these traumatic physical health consequences could be explained at the neighbourhood level.

## Discussion

This study assessed associations between IPV and traumatic physical health consequences among women 15 - 49 years old in Nigeria. IPV was found to be a common problem in Nigeria as reflected in the high prevalence, which is consistent with findings in Nigeria and elsewhere [3][47,48], confirming the first hypothesis in this study. This is not surprising when set against the backdrop of the pervasive patriarchal ideology in traditional societies such as the one in Nigeria; the widespread acceptance of gender roles and tolerance of certain levels of abuse is reflected in the reluctance of some abused women to seek medical care and/or crisis services [62], or the sometimes dismissive attitude among the Police on the premise that IPV is a family matter [63]. These findings however call for further studies on injury-related sequelae of IPV in order to better reflect the violence topography and permit more sensitive risk assessments.

That bruises, wounds, and severe burns were significantly associated with IPV is an expected finding given the high prevalence of IPV found in this study; previous studies reporting similar findings indicating that IPV by a current or former partner was the most common cause of injury to women [64,65]. Specifically, bruises were the most common of the traumatic physical consequences, and this findings is consistent with those in previous studies [9][64]. Though the aetiology of these consequences were not assessed due to absence of information on mechanisms or causes of such effects in the DHS data, bruises may arise from lesser amount of force, such as being slapped, compared to being struck with a fist or object [65].

The persistent and robust association between controlling behavior and IPV (within the bivariate and multivariate analyses), as well as with all the traumatic physical consequences examined in this study is a validation of recent finding of the strong relationship between controlling behavior and IPV within the same [20], and different study population [21,22], as well as a validation of the second hypothesis in this study. Thus, controlling behavior seems to play a significant role in the propensity to commit violent acts against women, most likely acts as predisposing factor to violence, although other factors may be needed to fully explain this relationship with IPV. This emphasizes the need for legal and social interventions to prevent and manage men's controlling behavior.

Being young (24 years or younger) appeared to be protective against sustaining wounds, and severe burns associated with IPV. This is an interesting finding that is inconsistent with other studies showing higher likelihood of IPV among younger women [66], further research is needed to examine the intertwined nature and severity of the traumatic physical consequences (wounds, and severe burns) with IPV. It is possible that a combination of factors, such as lower education and earnings, and lower decision-making autonomy associated with younger women may appear less threatening to certain more traditional men that propagate gender discriminatory roles and perceive themselves as dominant their wives.

The influence of women's education in this study could be seen protective. At the individual level, the protective influence of women's education on IPV and the likelihood of sustaining severe burns was proportionately stronger at higher levels of education, and is a corroboration of previous findings [67,68]. The association between a woman's education and risk of IPV and traumatic consequences can be explained through a number of mechanisms. With the relationship between IPV and economic disadvantage in Nigeria [16], individual education could provide a woman with knowledge and skills needed to improve her ability to manage a household despite reduced economic resources, thereby making her more resourceful within the household and decreasing her likelihood of IPV and traumatic consequences. Education could further present a woman with more opportunities for financial development and independence, allowing her to leave an abusive husband [69], and providing her husband/partner with an incentive to desist from abusing her. An educated wife may also be more valued and respected compared with an uneducated wife, thereby providing her further from abuse [70].

At the community level, the association between women's education and IPV was accounted for by women's community mean education being associated with a lower likelihood of sustaining wounds, and indicates the importance of women's educational context in relation to the likelihood of abuse. This is in agreement with previous studies suggesting that violence against women is associated with community attitudes accepting or are indifferent to IPV [25], and that the social norms underlying these attitudes vary with societal context [37]. Since education is an important factor in determining the acceptability of abuse [68], education at the community level may influence the likelihood of a woman being abused by acting through social norms to change community acceptability of such abusive acts, and positively influence institutional resources and interpersonal support against abuse towards women. By so doing, community-level education may interact with women's individual education, to reduce the likelihood of IPV. This study also found that women's justification of wife beating at the community level was associated with IPV and all the traumatic physical consequences examined in this study. Women's justification of wife beating at the community level represents their collective tolerance or acceptance of abuse and to some degree reflects the wider social norms, values and attitudes towards gender inequality within the community [67]. Justification of wife beating at the community level has been previously identified as an important mechanism underlying women's risk of IPV in different contexts [25][67,68], thus corroborating findings in this study. If women are to experience the full advantage of education in reducing their likelihood of IPV, there is a need to change community attitudes towards the acceptance of IPV, given that exposure to hostile behaviors in the community tends to directly influence the behaviors of husbands and wives within the family environment via a modeling process, these modeled hostile interaction styles and behaviors tend to be transferred into intra-marital interactions [71]. Thus, these findings call for policies that implement societal- and community-level public health measures aimed at changing community norms that justify control behavior and violence, as well as promoting normative environments that discourage residents' hostile behaviours by means of internalization of positive norms.

Gender norms in patriarchal societies such as Nigeria tolerate, legitimize or are at best indifferent to the use of violence against intimate partners under several circumstances. With men often socialized into violence, and aggressive behaviour often learned in the family, from peers and in the community, preventing the widespread use of controlling behaviours (including violence) against women by male partners may require initiatives at various levels, including at the national level, a national violence prevention strategy or movement that outlines a unified, coordinated and evidence-based national response to violence, spearheaded by the highest level of Government, involving several health and mental health professionals, and directed across all Departments of Government and civil society. With this strategy being culturally appropriate and community-based, especially among vulnerable socio-economic and ethnic groups, the main goal would have to be to achieve a systematic reduction in levels of all forms of control and violence, with a system for monitoring its progress incorporated within the healthcare sector - often the first point of call for abused women. Initiatives at the individual level should include aiming to change gender norms, commencing with children and adolescents (e.g. especially targeting boys in schools, so as to emphasize equality between sexes),moving on to primary prevention via all forms of media, secondary prevention through individual counselling, psychotherapy and social-casework with male and female victims, and perpetrators of violence. In addition, pro-active law enforcement should include persecution of perpetrators of violence, tertiary prevention through routine screening of women at healthcare visit, as well as treatment and rehabilitation of victims and perpetrators alike so as to prevent re-victimization (e.g. shelters for victims, anger management for perpetrators). These prevention efforts would however require unswerving political will, commitment and resources - without this, local efforts are bound to be ineffective.

### Strengths and limitations

The results of this study should be considered in the context of several limitations. The study used data based on the women's self-report of physical trauma following IPV, which may be subject to reporting bias. Disclosure rates of health consequences may not to be representative of the magnitude of the problem due to cultural factors [49]. The cross-sectional design limits the ability to draw causal inferences. The study assessed lifetime IPV and traumatic physical consequences, rather than IPV within the last 12 months. Administratively defined boundaries for neighbourhoods in this study may misclassify individuals into inappropriate administrative boundaries, therefore generating information bias. Some key strengths of this study are its use of a large sample size, the use of nationally representative country-level data, the simultaneous assessment of individual- and community-level factors, the extended scope of the traumatic physical consequences assessed, and the adherence to stringent ethical rules when collecting DHS data on domestic violence.

## Conclusions

IPV remains a problem of public health significance in Nigeria associated with bruises, wounds, and severe burns at individual level. Community-level factors such as education, and attitudes justifying wife beating exert significant influence on the likelihood of women to experience IPV and to sustain traumatic physical health consequences. The findings in this study provide evidence that exposure to IPV is associated with increased likelihood of traumatic physical consequences, and affirms the importance of women's individual contextual level education on IPV. Although our study cannot establish a causal relationship, it does suggest that paying greater attention to educating girls so as to protect them against IPV in adulthood, education women so as to empower them to bargain for better treatment or to leave abusive relationships, increase their economic resources, and educating men (who tend to perpetuate the violence), may reduce the likelihood of IPV and its traumatic physical consequences. Measures could also be aimed at creating social environments that are intolerant towards IPV, more difficult for men to perpetrate violence, and less difficult for women to report acts of intimate partner violence. It may be of interest to advocates of policies promoting inclusion of possible health consequences associated with IPV within healthcare screening, and interventions targeted at changing gendered societal- and community-level norms that justify control and violence, as well as preventing and managing controlling behavior within relationships.

## Competing interests

The authors declare that they have no competing interests.

## Authors' contributions

DA conceptualized the study, conducted the data analysis, wrote the original manuscript, carried out the revision of the subsequent drafts, and approved the final version of the manuscript.

## Pre-publication history

The pre-publication history for this paper can be accessed here:

http://www.biomedcentral.com/1472-6874/11/56/prepub
